# Endoscopic resection for gastric submucosal tumors: A single‐center experience in Japan

**DOI:** 10.1002/deo2.402

**Published:** 2024-07-15

**Authors:** Ippei Tanaka, Yuto Shimamura, Haruhiro Inoue, Daisuke Azuma, Kei Ushikubo, Kazuki Yamamoto, Hiroki Okada, Yohei Nishikawa, Mayo Tanabe, Manabu Onimaru

**Affiliations:** ^1^ Digestive Diseases Center Showa University Koto Toyosu Hospital Tokyo Japan

**Keywords:** endoscopic resection, endoscopic full‐thickness resection, endoscopic muscularis dissection, endoscopic subserosal dissection, gastric submucosal tumors

## Abstract

**Objectives:**

Endoscopic resection (ER) for gastric submucosal tumors (SMTs) has gained prominence in recent years, with studies emerging from various countries. However, there is a paucity of reports from Japan. We aimed to elucidate the efficacy and safety of ER for gastric SMT in Japan.

**Methods:**

In this retrospective observational study, we investigated the outcomes of consecutive patients who underwent ER for gastric SMT from January 2017 to May 2023. The outcome variables assessed included the complete resection rate, procedure time, closure‐related outcomes, and the incidence of adverse events.

**Results:**

A total of 13 patients were included in the analysis. The median procedure time was 163 (55–283) min. Complete full‐thickness resection was performed in seven cases, while in four cases, the serosa remained, and in two cases, the outer layer of the muscularis propria remained. In two cases where the SMT was located on the anterior side, conversion to laparoscopic surgery became necessary, resulting in a procedural success rate of 84.6% (11/13). Excluding these two cases, endoscopic closure of the defect was successfully accomplished in the remaining 11 cases. R0 resection was achieved in 12 out of 13 cases (92.3%). Although one patient had peritonitis, which was successfully treated conservatively, no other treatment‐related adverse events were encountered.

**Conclusions:**

Although ER for SMT on the anterior side may be challenging, our experience revealed that ER is a safe and efficacious approach for gastric SMT.

## INTRODUCTION

Gastric submucosal tumors (SMTs) represent one of the frequently encountered findings during upper endoscopy. The majority of SMTs, such as leiomyomas or schwannomas, are benign and often do not require treatment. However, in cases where there is evidence of enlargement or suspicion of gastrointestinal stromal tumors (GIST), surgical resection is considered,^1^ but there is a potential risk of removing excessive normal mucosa around the tumor resulting in gastric deformity.^2^ In addition, identifying the lesion during surgery can be challenging, and tumors located at the esophagogastric junction or pylorus pose particular difficulties during resection. To address these challenges, laparoscopic and endoscopic cooperative surgery (LECS) was introduced as a novel treatment, initially reported in Japan.^3^ LECS is currently considered the primary treatment for gastric SMTs in Japan, with its efficacy well‐documented in the literature.^4^, ^5^

Nevertheless, recent advancements in endoscopic technology and devices have led to increasing cases of endoscopic resection (ER) as a preferred method for treating SMTs. The initial concept of ER for gastric SMTs was reported in 2001.^6^ Despite numerous reports highlighting its high efficacy and safety, a majority of these studies are from China,^7^, ^8^, ^9^ with very few reports from Japan.^10^ Notably, ER has not gained widespread acceptance in Japanese clinical practice though it is covered as advanced medical care in Japan. In this study, we aimed to elucidate the clinical outcomes of ER for gastric SMTs.

## MATERIALS AND METHODS

This retrospective observational study was conducted at Showa University Koto Toyosu Hospital, a tertiary referral center in Tokyo, Japan. The clinical outcomes of consecutive patients who underwent ER for gastric SMTs between January 2017 and May 2023 were evaluated. Ethical approval for the study was obtained from the hospital's Institutional Review Board (Registration No.2023‐155‐B), following the principles of the Helsinki Declaration. All patients involved in the study provided written informed consent for the treatment procedure.

### Pre‐treatment examination

Prior to endoscopic treatment, a thorough pre‐treatment assessment was conducted for all participants in the study. Upper endoscopy and computed tomography scans were performed to identify the presence of gastric SMTs. Once the lesion was identified during endoscopy, endoscopic ultrasound sonography (EUS) was performed. When deemed feasible based on the size of the lesion, endoscopic ultrasound‐guided fine‐needle biopsy was utilized to obtain pathological findings prior to treatment. If the SMT was histologically diagnosed as a GIST, treatment was conducted. In cases where an increase in size or suspected malignant potential of the lesion was observed, treatment was planned without waiting for pre‐treatment pathological confirmation.

## TREATMENT STRATEGY

At our institution, the treatment strategy was determined based on the maximum diameter of SMTs. When the maximum diameter of the lesion exceeded 3 cm, surgical resection or LECS was selected because lesions exceeding 3 cm are generally not amenable to retrieval via the oral route. Conversely, for lesions measuring 3 cm or less, ER was typically selected as the primary treatment approach regardless of their location within the stomach. Furthermore, the basic indication for endoscopic treatment for gastric SMTs is an intraluminal growth pattern, but we also consider this treatment for extraluminal tumors if their position can be identified endoscopically and the tumor diameter is 3 cm or less. Patients who were initially considered for ER and converted to laparoscopic surgery were included in this study.

## ER PROCEDURE

All procedures were performed by a single expert endoscopist in an operating room under general anesthesia, utilizing a single‐channel endoscope (GIF‐290T; Olympus Corp.) with a super‐soft transparent hood (Space adjuster; TOP Corp.) attached. The electrosurgical generator VIO300D (Erbe Elektromedizin GmbH) was applied for the procedure. Mucosal incisions and submucosal dissections primarily utilized the Triangle Tip Knife J (KD‐645L; Olympus Corp.), with the IT knife 2 (KD‐611L; Olympus Corp.) being employed in cases where the endoscopic approach was challenging. CO_2_ insufflation was used in all procedures.

**FIGURE 1:**
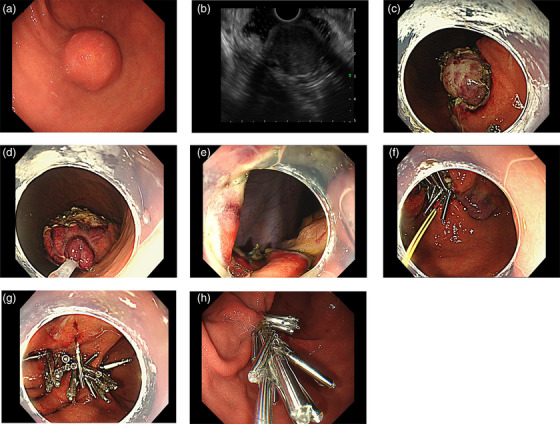
Representative case of endoscopic full‐thickness resection. (a) A submucosal tumor measuring 25 mm in size was located at the greater curvature of the lower gastric body. (b) Endoscopic ultrasonography showed a low echoic mass mixed with a slightly high echoic area, situated at the muscle layer. The lesion was suspected of a gastrointestinal tumor. (c) After the submucosal injection of hyaluronic acid, mucosal incision, and submucosal dissection was performed and the tumor was clearly seen. (d) Snare traction method was applied. (e) The defect after the tumor resection. (f) Loop11 closure method was applied to the large defect. (g) The defect was completely closed. (h) Two months after the procedure, the defect almost became scar.

The basic strategy of ER for gastric SMTs closely resembled the procedure in endoscopic submucosal dissection. Initially, a saline‐indigo carmine injection was administered around the target lesion, followed by mucosal incisions using the Triangle‐Tip Knife J. Mucosal incision was performed in EndoCut mode (Effect 2, Duration 3, Interval 3), while submucosal dissection was carried out utilizing spray coagulation mode (Spray coagulation mode, 50W, effect 2). Upon achieving sufficient submucosal dissection for visualizing the tumor surface, traction methods were implemented, utilizing two distinct techniques; multi‐point traction method and double‐scope traction technique. These two traction techniques are fully explained in the previous papers we have written.^11^, ^12^, ^13^ After achieving sufficient submucosal dissection around the lesion, muscle layer dissection was performed in the latter half of the procedure to minimize the time of full‐thickness resection. The representative case is illustrated in Figure 1.

**FIGURE 2:**
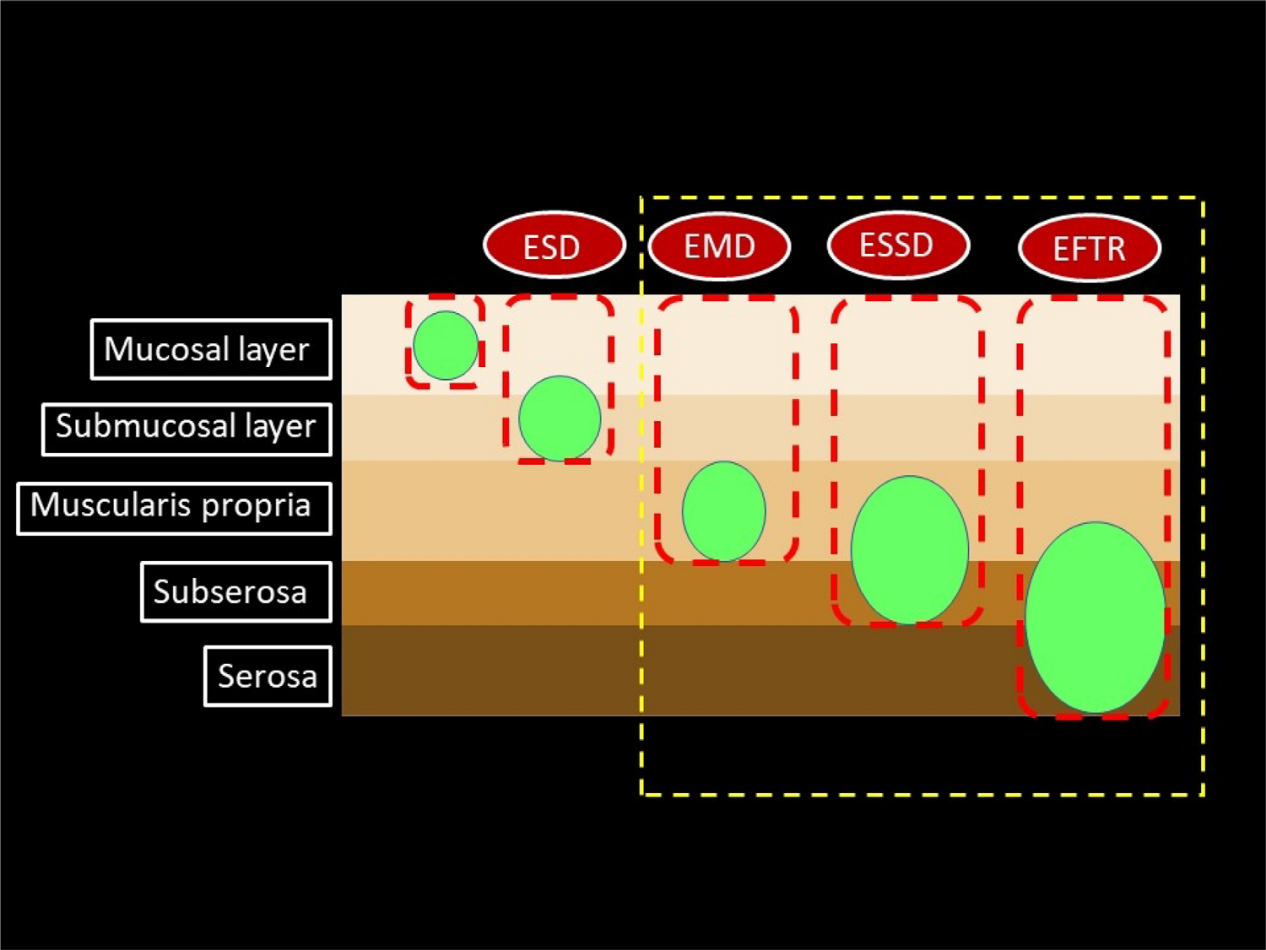
Schema of endoscopic submucosal dissection (ESD), endoscopic muscle dissection (EMD), endoscopic subserosa dissection (ESSD), and endoscopic full‐thickness resection (EFTR).

The therapeutic approach was classified into three distinct techniques based on the depth of the resected layer (Figure 2). Following the complete resection of the tumor if the outer layer of the muscularis propria remained, the procedure was classified as endoscopic muscle dissection (EMD). In cases where the serosa was preserved, it was classified as endoscopic subserosa dissection (ESSD). Conversely, if complete full‐thickness resection occurred, it was classified as endoscopic full‐thickness resection (EFTR).

### Mucosal defect management

**FIGURE 3:**
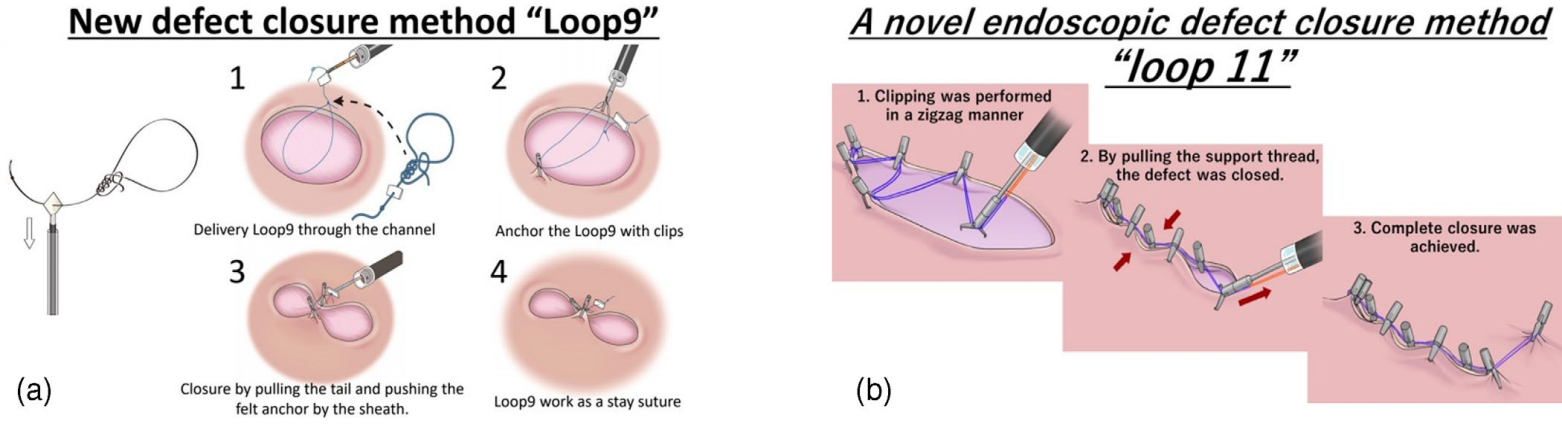
(a) Schema of Loop9. (b) Schema of Loop11.

The management of mucosal defects involved the application of a simple closure technique with endoclips. In scenarios where closure proved challenging, our institution's developed novel closure techniques were employed. Specifically, the Loop9 and Loop11 closure techniques, utilizing threads and multiple clips, were employed as necessary (Figure 3). A comprehensive description of this technique has been provided in a prior publication.^14^, ^15^, ^16^ Furthermore, a purse‐string suture method using an endoloop (HX‐400U‐30; Olympus) and endoclips was employed.^17^, ^18^ Additionally, the Mantis clip (Boston Scientific, M00521420) was strategically utilized to effectively close defects. This clip features a specialized anchor designed to securely grasp the defect edges of the defect, facilitating closure for defects measuring up to 3 cm in diameter.

The criteria for selecting these different methods are as follows: Loop11 was chosen for closing defects that were long in the longitudinal direction. Loop9 was used to approximate parts of large defects, with the remaining areas being closed with clips. Since the Mantis clip became commercially available, it has primarily been used for closure.

### Postoperative management after ER

The postoperative management approach varies based on the employed resection method, namely EMD, ESSD, or EFTR. For cases of EMD or ESSD, a second‐look endoscopy was conducted on the first postoperative day (POD 1) to evaluate potential occurrences of postoperative bleeding and leakage. Following the confirmation of the absence of adverse events, patients initiated fluid intake on the same day. By POD 2, diet resumption commenced, progressing based on individual tolerance, and patients were typically discharged on POD 5.

Conversely, for EFTR cases, fluid intake was initiated on POD 2. A second‐look endoscopy was conducted on POD 3, and if no adverse events were found, food intake commenced on POD 5, with discharge occurring on POD 8. To assess the healing of the tumor resection site, a follow‐up endoscopy was conducted four weeks after the resection. Subsequently, annual endoscopies were performed for surveillance, along with computed tomography scans if the lesion was diagnosed as GIST.

### Pathological diagnosis

All specimens were fixed in 10% formalin, and pathological diagnosis relied upon the utilization of hematoxylin and eosin staining, in conjunction with immunochemical findings. The lesion size was measured at their largest dimension. If the lesion was diagnosed as GIST, the mitotic count was assessed in 50 high‐power fields (HPFs), and the lesions were stratified into four distinct groups: “very low”, “low”, “intermediate”, and “high‐risk”. This classification framework adhered to the modified Fletcher classification.^19^

### Outcomes and definitions

The primary endpoint involved the R0 resection rate, defined as en bloc resection characterized by a microscopically verified clear margin. Conversely, R1 was defined as a positive pathological margin, capsule pathological injury, or piecemeal resection. Secondary outcomes included the procedural success rate, signifying the proportion of cases in which the endoscopic procedure concluded with successful defect closure without necessitating conversion to surgical intervention. Procedure time was defined as the duration elapsed from the start of submucosal injection to the completion of defect closure. Adverse events included postoperative bleeding, leakage, and peritonitis. The hospitalization period was defined as the duration from ER to discharge.

Furthermore, ER cases in this study were divided into two groups based on the depth of the resected layer: the EMD/ESSD group and the EFTR group. Clinical outcomes were then compared. Continuous data were expressed as the median and interquartile range (IQR) and compared using the Mann–Whitney U test.

## RESULTS

### Patient baseline characteristics

In this study, ER was performed on 13 cases where the tumor diameter was 3 cm or less. On the other hand, five cases with SMTs larger than 3 cm during the study period were deemed suitable for LECS treatment. The background characteristics of the 13 patients with gastric SMT are detailed in Tables 1 and 2. The gender distribution was observed as six males to seven females, with a median age of 63 years (range: 48–76). None of the patients were on anticoagulant drugs. The median size of the tumors was 15 mm (7–22), with six tumors located in the upper third of the stomach, three in the middle third, and four in the lower third. Two lesions were located on the anterior side. Ten lesions (76.9%) exhibited an intraluminal growth pattern. Endoscopic ultrasound sonography was performed in 12 out of 13 patients, with endoscopic ultrasound sonography‐fine‐needle aspiration conducted in nine cases. The rationale for resection included histological confirmation of GIST in six patients and tumor enlargement in the remaining seven patients.

**TABLE 1:** Summary of the patient who underwent endoscopic muscle dissection (EMD) or endoscopic subserosa dissection (ESSD).

No.	Age	Sex	Anticoagulant	Tumor size	Tumor location	Location	Growth type	Treatment method
1	60	F		8	U	Post	Intra	ESSD
2	75	F		20	U	Gre	Intra	EMD
3	54	F		18	M	Less	Intra	ESSD
4	73	F		15	U	Post	Extra	ESSD
5	66	M		7	U	Post	Extra	ESSD
6	63	M		13	L	Gre	Intra	ESSD

Traction method	Closure method	Procedure time	Closure time	Abdominal puncture	Hospital stay	Pathological diagnosis	R0 resection	Follow‐up period	Recurrence
Snare	Clip with microloop	55	20	‐	10	Leiomyoma	R0	3	‐
Snare	Loop9	120	40	‐	6	GIST (low)	R0	7	‐
Snare	Microring	106	35	‐	4	GIST (low)	R0	15	‐
Snare	Loop9	99	40	‐	5	GIST (low)	R0	17	‐
Double scope	Loop9	130	43	‐	4	Leiomyoma	R0	2	‐
Double scope	Loop9	100	24		5	Inflammatory granuloma	R0	4	‐

Abbreviations: EMD, endoscopic muscle dissection; ESSD, endoscopic subserosa dissection; EFTR: endoscopic full‐thickness resection; IQR, interquartile range.

**TABLE 2:** Summary of the patient who underwent endoscopic full‐thickness resection.

No.	Age	Sex	Anticoagulant	Tumor size	Tumor location	Location	Growth type	Conversion to surgery
1	68	F	‐	15	U	Post	Intra	‐
2	48	F	‐	18	U	Gre	Intra	‐
3	53	M	‐	15	M	Ant	Intra	+
4	65	F	‐	19	M	Less	Extra	‐
5	58	M	‐	13	L	Post	Intra	‐
6	76	M	‐	22	L	Ant	Intra	+
7	52	M	‐	20	L	Gre	Intra	‐

Traction method	Closure method	Procedure time	Closure time	Abdominal puncture	Adverse events	Hospital stay	Pathological diagnosis	R0 resection	Follow‐up period	Recurrence
Snare	Purse‐string suturing	176	50	‐		10	GIST (low)	R0	36	‐
Snare	Loop9	141	60	+		7	GIST (low)	R0	16	‐
Snare	Surgical closure	291		‐		7	Schwannoma	R0	2	‐
Double scope	Loop9	149	35	+		7	GIST (low)	R0	9	‐
Double scope	Loop9	189	40	‐		8	GIST (low)	R1	11	‐
Snare	Surgical closure	283				8	I GIST (low)	R0	8	‐
Snare	Loop9+11	280	100	+	Peritonitis	12	Schwannoma	R0	2	

Abbreviation: EFTR, endoscopic full‐thickness resection.

### Procedural and clinical outcomes

The procedural outcomes and any associated adverse events are presented in Tables 1 and 2. Therapeutic interventions for gastric SMT included EMD in one patient, ESSD in five patients, and EFTR in seven patients. The median procedural time was 141 min (55–283), including a median closure time of 40 min (20–100). In two cases where the SMT was located on the anterior side, conversion from endoscopic to laparoscopic surgery was required, resulting in a procedural success rate of 84.6% (11/13). Excluding these two cases, endoscopic closure of the defect was successfully accomplished in the remaining 11 cases. R0 resection was achieved in 12 out of 13 cases (92.3%). Closure techniques employed the thread and clip closure method (Loop9 or Loop 11) in eight cases, micro‐ring in two cases, and a purse‐string suture method in one case. Although one patient suffered from peritonitis, which was treated conservatively, the others also showed no treatment‐related adverse events. The median duration of hospital stay was 7 days (4–12), and a follow‐up endoscopy conducted 2 months after treatment revealed complete healing of the defect. During the median follow‐up period of 11 months (2–36), no metastatic recurrence was observed.

Pathological diagnoses included GIST in eight patients (61.5%), all of whom had a very low‐grade risk. Schwannoma was identified in two cases (15.3%), leiomyoma in one case (7.7%), and granuloma in one case (7.7%).

Regarding two cases of conversion to surgery, following the full‐layer resection, a leakage of gastric air into the abdominal cavity occurred, causing endoscopic invisibility. This rise in intra‐abdominal pressure also resulted in an increase in thoracic pressure, exacerbating respiratory conditions. Thus, a transition was made to laparoscopic treatment, which ultimately facilitated a successful R0 resection without any postoperative complications.

### Comparison of clinical outcomes between EMD/ESSD and EFTR

Clinical outcomes between the EMD/ESSD group and EFTR group are represented in Table 3. The median procedure time in the EMD/ESSD group was significantly shorter than that in the EFTR group (103 vs. 189 min, *p*‐value < 0.05). Although the highest white blood cell count (WBC) and body temperature were higher in the EFTR group compared to the EMD/ESSD group, a significant difference was not observed. The median hospital stay in the EFTR group was significantly longer than that in the EMD/ESSD group (8 days vs. 6 days, *p* < 0.05).

**TABLE 3:** Treatment outcomes between the endoscopic muscle dissection (EMD)/endoscopic subserosa dissection (ESSD) group and endoscopic full‐thickness resection (EFTR) group.

	EMD/ESSD group *n* = 6	EFTR group *n* = 7	*p*‐value
Procedure time, min, median (IQR)	103 (99–120)	189 (149–283)	<0.05
Highest WBS, median (IQR)	8480 (6240–10,150)	11,140 (8960–14,770)	0.169
Highest body temperature, median (IQR)	37.0 (36.9–37.1)	37.4 (36.8–37.7)	0.438
Hospital stay, days, median (IQR)	5 (4‐6)	8 (7–10)	<0.05

Abbreviations: EMD, endoscopic muscle dissection; ESSD, endoscopic subserosa dissection; EFTR, endoscopic full‐thickness resection; IQR, interquartile range.

Although the highest values of WBC and body temperature were not significantly different, we also investigated the changes in these parameters. No significant changes were observed in the EMD/ESSD group between POD0 and POD3. However, a notable increase in body temperature and WBC was seen on POD1 in the EFTR group. Nevertheless, these parameters showed improvement on POD3.

## DISCUSSION

Endoscopic resection for gastric SMTs was classified into three types based on the depth of resection: EMD, ESSD, and EFTR. In this study, we compared clinical outcomes between two groups: the EMD/ESSD group and the EFTR group. The median procedure time and hospital stay in the EFTR group were significantly longer than those in the EMD/ESSD group. The extended procedure time in the EFTR group can be attributed to the full‐layer resection involved, which introduces a higher level of complexity in endoscopic techniques, particularly in the closure technique. A secure closure of the defect is required to prevent peritonitis, which can contribute to the longer procedure time in EFTR. Furthermore, the hospital stay in the EFTR group was longer because considering the clinical course of EFTR cases, the patient's body temperature and WBC increased right after the treatment, and a longer period of fasting was needed. However, these parameters showed improvement as early as on POD3, allowing the initiation of a diet. Though it is impossible to predict preoperatively whether the resection will result in EMD, ESSD, or EFTR, our study shows that the EFTR group follows a clinical course nearly identical to the EMD/ESSD group. This indicates that EFTR is safe as long as a secure closure method is applied.

In this study, there were two cases in which ER was converted to laparoscopic surgery. The primary cause for this transition from ER to surgery was the loss of visibility during the endoscopic procedure. In both cases, after the full‐layer resection, gastric air flowed into the abdominal cavity, resulting in impaired visibility of the endoscopic procedure. Given the difficulty in continuing ER, the conversion from ER to laparoscopic treatment was done, resulting in successful R0 resection, with no postoperative adverse events. The cause of impaired endoscopic visibility in both cases is believed to be the presence of lesions at the anterior wall of the stomach. Past reports have also indicated that when lesions are located at the anterior wall of the stomach, completing the EFTR technique and closing the defect can become extremely challenging.^20^, ^21^ Therefore, when performing ER for gastric SMTs located on the anterior wall, it is essential to develop a treatment strategy in which full‐thickness resection ia performed as late as possible during the procedure and to have close collaboration with a surgeon.

Various methods have been reported for closing post‐resection defects after ER. Commonly known methods include simple clipping, purse‐string sutures,^17^, ^18^ and over‐the‐scope clips.^22^, ^23^ In our institution, we employ a closure method using threads and clips referred to as Loop9 and Loop11. For a detailed description of this technique, we refer readers to other publications.^6^, ^7^, ^8^, ^9^, ^10^, ^11^, ^12^, ^13^, ^14^ In all cases of this study, complete closure was achievable, and there were no closure‐related adverse events, such as air leakage or delayed perforation. These closure techniques were deemed highly effective. It is essential to adapt and choose the appropriate technique based on the clinical situation, as employing a variety of techniques is necessary for successful management.

Although a multicenter study on ER for gastric SMTs was conducted previously,^24^ our study has two strengths. First, we provide a detailed explanation of the various resection and closure methods used at our institution and elucidate the detailed clinical course for each case. Second, we investigate whether differences in resection depth affect clinical outcomes.

While this case series from Japan provides valuable insights into real‐world clinical outcomes, its major limitation lies in being a single‐center retrospective study. Additionally, all procedures in this study were carried out by an expert endoscopist. Consequently, larger‐scale, multi‐center studies may be essential to assess its broader applicability.

In conclusion, ER for gastric SMTs is considered a safe and viable treatment option. However, it is essential to establish a reliable method for closing defects, conduct procedures in an operating room, and have a close collaboration with surgeons to ensure a seamless transition to surgery when deemed necessary.

## CONFLICT OF INTEREST STATEMENT

Author Haruhiro Inoue is an advisor for Olympus Corporation and Top Corporation. He has also received educational grants from Olympus Corporation and Takeda Pharmaceutical Co. The other authors declare no conflict of interest.

## ETHICS STATEMENT

‐Approval of the research protocol by an Institutional Reviewer Board. Ethical approval for the study was obtained from the hospital's Institutional Review Board (Registration No.2023‐155‐B), following the principles of the Helsinki Declaration.

‐Informed Consent. All patients involved in the study provided written informed consent for the treatment procedure.

‐Registry and the registration no. of the study/trial. N/A.

‐Animal Studies. N/A.
